# Dynamic Stability Analysis in Hybrid Nanocomposite Polymer Beams Reinforced by Carbon Fibers and Carbon Nanotubes

**DOI:** 10.3390/polym13010106

**Published:** 2020-12-29

**Authors:** Behrooz Keshtegar, Reza Kolahchi, Arameh Eyvazian, Nguyen-Thoi Trung

**Affiliations:** 1 Division of Computational Mathematics and Engineering, Institute for Computational Science, Ton Duc Thang University, Ho Chi Minh City 800010, Vietnam; beh.keshtegar@tdtu.edu.vn (B.K.); nguyenthoitrung@tdtu.edu.vn (N.-T.T.); 2 Faculty of Civil Engineering, Ton Duc Thang University, Ho Chi Minh City 800010, Vietnam; 3 Institute of Research and Development, Duy Tan University, Da Nang 550000, Vietnam; 4 Mechanical and Industrial Engineering Department, College of Engineering, Qatar University, P.O. Box 2713 Doha, Qatar

**Keywords:** polymer beam, carbon fibers, carbon nanotubes, dynamic stability, exponential shear deformation beam theory

## Abstract

The objective of this innovative research is assessment of dynamic stability for a hybrid nanocomposite polymer beam. The considered beam formed by multiphase nanocomposite, including polymer–carbon nanotubes (CNTs)–carbon fibers (CFs). Hence, as to compute the effective material characteristics related to multiphase nanocomposite layers, the Halpin–Tsai model, as well as micromechanics equations are employed. To model the structure realistically, exponential shear deformation beam theory (ESDBT) is applied and using energy methods, governing equations are achieved. Moreover, differential quadrature method (DQM) as well as Bolotin procedures are used for solving the obtained governing equations and the dynamic instability region (DIR) relative to the beam is determined. To extend this novel research, various parameters pinpointing the influences of CNT volume fraction, CFs volume percent, boundary edges as well as the structure’s geometric variables on the dynamic behavior of the beam are presented. The results were validated with the theoretical and experimental results of other published papers. The outcomes reveal that increment of volume fraction of CNT is able to shift DIR to more amounts of frequency. Further, rise of carbon fibers volume percent leads to increase the excitation frequency of this structure.

## 1. Introduction

When a structure is subjected to periodic and pulsatile loads, it is well known that the ordinary forced response will lead to dynamic instability. This subject is very important since the periodic loads may cause parametric vibrations, which may damage the structures. Nanocomposite plates can be used in different industries such as aircrafts and automobile which may be subjected to periodic and pulsatile loads. However, dynamic stability of nanocomposite plates is a novel topic which should be studied.

Different papers were brought into investigation regarding polymer nanocomposites (PNCs) special applications [1], device usages [2], its processing technologies for future usages [3,4,5,6] and safety analysis [7,8,9]. Pastoriza-Santos et al. [10] discussed the processes of manufacturing and different usages of plasmonic PNC ranging from light-harvesting improvement to tracing biologic as well as chemicals molecules. Leon et al. [11] brought up significant proficiency of PNC in terms of additive manufacturing usages. It is found that PNC are preferable due to their significant mechanical, chemical and thermal characteristics under harsh conditions. In the aspect of energy density, Liu et al. [12] analyzed dielectric PNC having multilayered structure. They claimed that the discharged energy density of this multilayered structure is the greatest discharged energy densities which were presented. Nevertheless, there are limitations in the conduction of polymers that Abbasi et al. [13] discussed and offered some advices. It is believed that there are constraints related to electrically conductive polymers and considering carbon-based nanoparticles can push back them. Hence, this research reviewed some improvements in this field.

Further, it should be noticed that hybrid composites refer to specific substances made up of organic and inorganic ingredients and nanocomposite term, points at hybrid inorganic–organic composites having components in nano scale. These materials are utilized in formations of structures because of their striking properties such as safety [14,15,16] and reliability [17,18,19]. Xia and Lo [20] offered a method by which ibuprofen (IBU)—considered as organic pollutant in flowing waters, can be eliminated from the water using hybrid nanocomposites. It is shown that hybrid nanocomposite matrix can be increased the strength of organic pollutant. Filippov et al. [21] presented a novel method for characterizing hybrid nanocomposites. Ebrahimi and Dabbagh [22] carried out a research regarding vibration of hybrid nanocomposite plates. Using classical plate theory and Hamilton principle, the governing equations are obtained and solved analytically. It is observed that hybrid nanocomposites are able to resist high amounts of frequency compared to the ordinary composites. Dabbagh et al. [23] reported thermal buckling of hybrid nanocomposites beam. Using refined plate theory as well as Hamilton’s principle, the governing equations are obtained and utilizing Galerkin’s procedure, the motion equations are solved. It is shown that hybrid nanocomposite structures are tougher toward critical buckling loads than conventional composites. Moreover, creep as well as viscoelastic behaviors of hybrid glass/epoxy nanocomposites are studied experimentally by Salehi and colleague [24].

Without doubt, besides invented methods as to tightening structures in various domains, reinforcing structures via nanocomposites and CFs have been featured strongly since last decade. Nanocomposites such as CNTs have been preferable materials to be added into structures for this intention due to high fracture resistance and capability of vibration damping. Regardless of nanotube’s types containing single walled carbon nanotubes (SWCNTs) and multi walled carbon nanotubes (MWCNTs) and their branches, they possess notable properties in different fields such as significant electrical and thermal conductivities, strength and elasticity and energy storage that have made them desirable. Moreover, investigations regarding CNTs and their composites are of interest between scientists to enhance damping properties of these materials, especially epoxy, in terms of loss modulus [25,26] or damping ratio [27,28]. There are researches exploring properties of nanocomposites Vinson [29] and Herman et al. [30]. Gul and Aydogdu [31] investigated wave dispersion of DWCNTs. They explained that, through Doublet Mechanics, the wave dispersion was studied which had precise outcomes for flexural as well as axial wave dispersion in nanotubes. Vibration analysis of SWCNTs was carried out by Avramov [32]. Using Sanders–Koiter shell methods—as well as nonlocal elasticity—the motion equations were derived and solved numerically via the Galerkin procedure. In another study, Bian and Wang [33] carried out a research as to buckling of DWCNTs in a thermal environment. The influence of finite temperature and various nonlocal effects have been studied. They revealed that buckling of DWCNT declines whereas the finite temperature rises. Natural frequency analysis of FG-CNTs curved shell panel which is shallow has been done by Mehar et al. [34]. In this study, higher-order theory is used for obtaining governing equations and finite element method (FEM) is utilized for solving equations. Further, the precision of this numerical method is compared with different numerical procedures. Zhang et al. [35] analyzed deflection of FG-CNTRC plate which is on the Pasternak medium. First-order shear deformation theory (FSDT) besides Ritz methods are utilized in order to obtain and solve governing equations, respectively. In this work, the influences relevant to CNT distributions and volume fraction and foundation up deflection are analyzed. Jiao et al. [36] discussed dynamic buckling behavior of FG-CNTRC shell. The structure is under time-varying load and FSDT is applied to derive motion equations. Galerkin method is used for solving the equations and the influence of dispersion of CNTs and their volume fraction and other parameters are taken into account. Mirzaei and Kiani [37] investigated buckling evaluation of FG-CNTRC plates in thermal environment. In this study, besides various parameters including different boundary conditions and aspect ratio, the influence of CNTs are discussed. They indicated that FG-X distribution type is the better choice. In another research, wave dispersion of FG nanocomposites which are reinforced through CNTs are investigated by Janghorban and Nami [38]. Dynamic analysis of structures induced by non-conservative loading and electromagnetic interactions has been studied in the literature. Based on the equivalent single-layer linear theory for laminated shells, Kolahchi et al. [39] presented dynamic buckling of sandwich nanoplate subjected to harmonic compressive load based on nonlocal elasticity theory. Mikhasev et al. [40] investigated free and forced vibrations of thin cylindrical sandwich panels with magnetorheological core. Hajmohammad et al. [41] analyzed dynamic response of sandwich plates under the blast load based on sinusoidal shear deformation theory. Malikan and Eremeyev [42] investigated the effect of flexoelectricity on dynamic response of piezoelectric nanobeam assuming internal viscoelasticity. Malikan et al. [43] studied torsional stability of a nanocomposite shell subjected to magnetic field utilizing nonlocal strain gradient theory. Keshtegar et al. [44] presented dynamic stability of nanocomposite-truncated sandwich conical shells based on differential cubature method.

To date, no research has been conducted studying and analyzing the dynamic behavior of a hybrid nanocomposite polymer beam reinforced via CFs as well as CNTs using ESDBT. In this study, we examine the beams composed by multiphase nanocomposites, including carbon/fiber/CNT/ polymer which the responses of beams are extracted using micromechanics—as well as Halpin–Tsai equations. With respect to DQM and Bolotin procedures, the obtained motion equations are solved, and DIR are attained, respectively. Distinct variables are investigated, such as the influences of CNT volume fraction, CF volume percent, boundary edges as well as the structure’s geometric variables on the dynamic behavior of the beam.

## 2. Problem Definition

**Figure 1:**
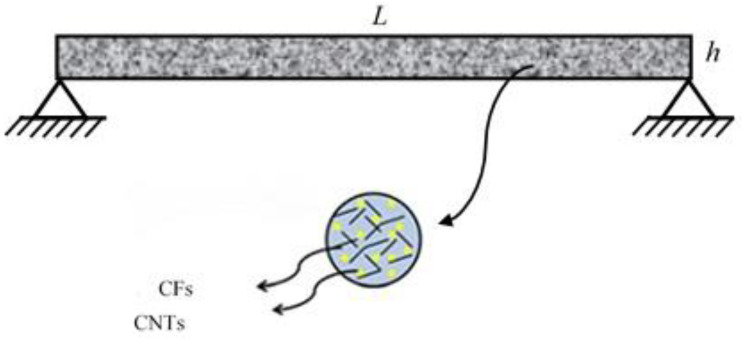
Configuration of polymer beam reinforced via carbon fibers (CFs) as well as carbon nanotubes (CNTs).

Figure 1 illustrates a polymer beam, in which the CNTs and CFs are considered the reinforcements. Further, the thickness and length of the beam, respectively are h and L.

In this section, in order to model the structure mathematically, ESDBT is employed and hence, the displacement vectors are given by [45]:

(1) $$\begin{array}{l} u_{1}(x,z,t)=u(x,t)-z\frac{\partial w(x,t)}{\partial x}+\Phi (z)\left(\frac{\partial w(x,t)}{\partial x}-\psi (x,t)\right),\\ u_{2}(x,z,t)=0,\\ u_{3}(x,z,t)=w(x,t). \end{array}$$

In above relation, $u_{1}$ and $u_{3}$ respectively describe mid-plane displacements in longitudinal and thickness directions. Moreover, ψ defines rotation related to cross section and Φ(z) represents shape function of the beam that can be written as

(2) $$\Phi (z)=ze^{-2{\left(z/h\right)}^{2}},$$

in which h refers to thickness of structure. Furthermore, the strain components of this structure are described as

(3) $$\varepsilon_{xx}=\left(\frac{\partial u}{\partial x}\right)-z\left(\frac{\partial^{2}w}{\partial x^{2}}\right)+\left(ze^{-2{\left(z/h\right)}^{2}}\right)\left(\frac{\partial^{2}w}{\partial x^{2}}-\frac{\partial \psi }{\partial x}\right),$$

(4) $$\gamma_{xz}=\left(e^{-2{\left(z/h\right)}^{2}}-\frac{4z^{2}e^{-2{\left(z/h\right)}^{2}}}{h^{2}}\right)\left(\frac{\partial w}{\partial x}-\psi \right).$$

It should be noted that stress–strain relations can be written as

(5) $$\sigma_{xx}^{}=C_{11}\varepsilon_{xx},$$

(6) $$\tau_{xz}^{}=C_{44}\gamma_{xz},$$

where $C_{11}$ and $C_{44}$ express the elastic constants achieved through Halpin–Tsai model.

### 2.1. Modeling CNT/Fiber/Polymer Multiphase Nanocomposite

Carbon nanotubes were grown directly on carbon fibers using chemical vapor deposition. When embedded in a polymer matrix, the change in length scale of carbon nanotubes relative to carbon fibers results in a multiscale composite [46]. Incorporation of micromechanics as well as Halpin–Tsai model [47] are utilized for reaching polymer beam’s equivalent material properties in two phases. Orthotropic effective characteristics related to CNT-reinforced multi-phase laminates are given by

(7) $$E_{11}=V_{F}^{}E_{11}^{F}+V_{MNC}^{}E_{}^{MNC},$$

(8) $$\frac{1}{E_{22}}=\frac{1}{E_{22}^{F}}+\frac{V_{MNC}^{}}{E_{}^{MNC}}-V_{F}^{}V_{MNC}^{}-\frac{\frac{\nu_{F}E_{}^{MNC}}{E_{22}^{F}}+\frac{\nu_{MNC}^{2}E_{22}^{F}}{E_{}^{MNC}}-2\nu_{F}\nu_{MNC}^{}}{V_{F}^{}E_{22}^{F}+V_{MNC}^{}E_{}^{MNC}},$$

(9) $$\frac{1}{G_{12}}=\frac{V_{F}^{}}{G_{12}^{F}}+\frac{V_{MNC}^{}}{G_{}^{MNC}},$$

(10) $$\rho =V_{F}^{}\rho_{}^{F}+V_{MNC}^{}\rho_{}^{MNC},$$

(11) $$\nu_{12}=V_{F}^{}\nu_{}^{F}+V_{MNC}^{}\nu_{}^{MNC},$$

in which G,E and ρ respectively express shear modulus, Young’s modulus and mass density; V as well as ν represent volume fraction and Poisson’s ratio, respectively. Further, the subscript and superscript *MNC* and *F,* respectively refer to matrix of nanocomposite and fibers. With respect to Halpin–Tsai equations, nanocomposite’s elastic modulus is written as follow [48]:

(12) $$E_{}^{MNC}=\frac{E_{}^{M}}{8}\left[5\left(\frac{1+2\beta_{dd}V_{CN}}{1-\beta_{dd}V_{CN}}\right)+3\left(\frac{1+2(\ell^{CN}/d^{CN})\beta_{dl}V_{CN}}{1-\beta_{dl}V_{CN}}\right)\right],$$

in which

(13) $$\beta_{dl}=\left(\frac{\left(E_{11}^{CN}/E_{}^{M})-(d^{CN}/4t^{CN}\right)}{\left(E_{11}^{CN}/E_{}^{M})+(\ell^{CN}/2t^{CN}\right)}\right),$$

(14) $$\beta_{dd}=\left(\frac{\left(E_{11}^{CN}/E_{}^{M})-(d^{CN}/4t^{CN}\right)}{\left(E_{11}^{CN}/E_{}^{M})+(d^{CN}/2t^{CN}\right)}\right),$$

in which $V_{M}^{}$ as well as $E_{}^{M}$ respectively describe matrix’s volume fraction and Young’s modulus. Moreover $V_{CN}^{}$, $E_{}^{CN}$, $t_{}^{CN}$, $d_{}^{CN}$ and $\ell_{}^{CN}$ respectively define volume fraction, Young’s modulus, thickness, outer diameter and length correlative to CNTs. The CNT volume fraction is given by

(15) $$V_{CN}^{}=\frac{w_{CN}}{w_{CN}+\left(\rho^{CN}/\rho^{m}\right)-\left(\rho^{CN}/\rho^{m}\right)w_{CN}},$$

in which $\rho^{m}$ and $\rho^{CN}$ respectively hint at mass density of matrix and CNTs and $w_{CN}$ is mass fraction. Likewise, Poisson’s ratio, mass density and shear modulus related to *MNC* are written as below:

(16) $$\nu_{}^{MNC}=\nu_{}^{M},$$

(17) $$\rho_{}^{MNC}=V_{CN}^{}\rho_{}^{CN}+V_{M}^{}\rho_{}^{M},$$

(18) $$G^{MNC}=\frac{E_{}^{MNC}}{2(1+\nu^{MNC})},$$

in which $\nu_{}^{MNC}$ as well as $\nu_{}^{M}$ respectively represent Poisson’s ratio related to *MNC* and matrix. It is mentioned that because of the CNT’s small extent, the Poisson’s ratio related to matrix and *MNC* will be assumed analogous [49].

### 2.2. Motion Equations

In this part, the structure’s strain energy is described as below:

(19) $$U=\frac{1}{2}\int_{V_{}}\left(\sigma_{xx}^{}\varepsilon_{xx}+\tau_{xz}^{}\gamma_{xz}\right)dV,$$

Likewise, by introducing Equations (3) and (4) into Equation (19), we have

(20) $$U=\frac{1}{2}\int_{0}^{L}[\int_{}[N_{x}^{}\left(\left(\frac{\partial u}{\partial x}\right)\right)-M_{x}^{}\left(\frac{\partial^{2}w}{\partial x^{2}}\right)+F_{x}^{}\left(\frac{\partial^{2}w}{\partial x^{2}}-\frac{\partial \psi }{\partial x}\right)+Q_{x}^{}\left(\left(\frac{\partial w}{\partial x}-\psi \right)\right)]dx,$$

Therefore, stress resultants are given by

(21) $$N_{x}=\int_{}^{}\sigma_{xx}^{}dA,$$

(22) $$M_{x}=\int_{}^{}\sigma_{xx}^{}zdA,$$

(23) $$F_{x}=\int_{}^{}\sigma_{xx}^{}\Phi (z)dA,$$

(24) $$Q_{x}=\int_{}^{}\tau_{xz}^{}\frac{\partial \Phi (z)}{\partial z}dA,$$

In order to extend above equations, Equations (3)–(6) are substituted into Equations (21)–(24) and the outcomes are presented in Appendix A. For the next step, the kinetic energy for this structure are written as

(25) $$K=\frac{\rho }{2}\int \left({\dot{u}}_{1}{}^{2}+{\dot{u}}_{2}{}^{2}+{\dot{u}}_{3}{}^{2}\right)dV$$

Substituting Equation (1) into Equation (25) yields

(26) $$K=\frac{\rho }{2}\int_{}^{}\left({\left(\frac{\partial u}{\partial t}-z\frac{\partial^{2}w}{\partial x\partial t}+z\left(\frac{\partial^{2}w}{\partial x\partial t}-\frac{\partial \psi }{\partial t}\right)\right)}^{2}+{\left(\frac{\partial w}{\partial t}\right)}^{2}\right)dV.$$

in which ρ refers to the density of the beam. The inertia moments are expressed as follow

(27) $$\left\{\begin{array}{l} I_{0}\\ I_{1}\\ I_{2}\\ I_{3}\\ I_{4}\\ I_{5} \end{array}\right\}=\int_{}^{}\left[\begin{array}{l} \rho \\ \rho z\\ \rho z^{2}\\ \rho \Phi (z)\\ \rho z\Phi (z)\\ \rho \Phi {\left(z\right)}^{2} \end{array}\right]dA,$$

Factoring in Equation (27), the Equation (26) is rewritten as

(28) $$\begin{array}{l} K=0.5\int_{}^{}[I_{0}\left({\left(\frac{\partial u}{\partial t}\right)}^{2}+{\left(\frac{\partial w}{\partial t}\right)}^{2}\right)-2I_{1}\left(\frac{\partial u}{\partial t}\frac{\partial^{2}w}{\partial x\partial t}\right)+I_{2}{\left(\frac{\partial^{2}w}{\partial x\partial t}\right)}^{2}\\ +I_{3}\frac{\partial u}{\partial t}\left(\frac{\partial^{2}w}{\partial x\partial t}-\frac{\partial \psi }{\partial t}\right)-I_{4}\frac{\partial^{2}w}{\partial x\partial t}\left(\frac{\partial^{2}w}{\partial x\partial t}-\frac{\partial \psi }{\partial t}\right)+I_{5}{\left(\frac{\partial^{2}w}{\partial x\partial t}-\frac{\partial \psi }{\partial t}\right)}^{2}]\;\;dx. \end{array}$$

Eventually, motion equations are achieved utilizing Hamilton’s principle:

(29) $$\int_{0}^{t}(\delta U-\delta K)dt=0,$$

By introducing Equations (20), (28) into Equation (29), the governing equations are expressed as follow

(30) $$\delta u:\frac{\partial N_{x}}{\partial x}=I_{0}\frac{\partial^{2}u}{\partial t^{2}}+\left(I_{3}-I_{1}\right)\frac{\partial^{3}w}{\partial x\partial t^{2}}-I_{3}\frac{\partial^{2}\psi }{\partial t^{2}},$$

(31) $$\begin{array}{l} \delta w:\frac{\partial^{2}M_{x}}{\partial x^{2}}+\left(2e_{31}V_{0}+P\right)\frac{\partial^{2}w}{\partial x^{2}}-\frac{\partial^{2}F_{x}}{\partial x^{2}}+\frac{\partial Q_{x}}{\partial x}=I_{0}\frac{\partial^{2}w}{\partial t^{2}}\\ +\left(I_{1}-I_{3}\right)\frac{\partial^{3}u}{\partial x\partial t^{2}}+\left(2I_{4}-I_{2}-I_{5}\right)\frac{\partial^{4}w}{\partial x^{2}\partial t^{2}}+\left(I_{5}-I_{4}\right)\frac{\partial^{3}\psi }{\partial x\partial t^{2}}, \end{array}$$

(32) $$\delta \psi :Q_{x}-\frac{\partial F_{x}}{\partial x}=I_{5}\frac{\partial^{2}\psi }{\partial t^{2}}-I_{3}\frac{\partial^{2}u}{\partial t^{2}}+\left(I_{4}-I_{5}\right)\frac{\partial^{3}w}{\partial x\partial t^{2}},$$

where $P=\alpha P_{cr}+\beta P_{cr}\cos(\omega t)$ in which α, β, $P_{cr}$ and ω are static load factor, dynamic load factor, critical load and frequency, respectively. For expansion of above equations, Equations (A1)–(A4) are substituted into Equations (30)–(32) and hence, they are expressed as below:

(33) $$\begin{array}{l} A_{11}\left(\frac{\partial^{2}u}{\partial x^{2}}\right)-B_{11}\left(\frac{\partial^{3}w}{\partial x^{3}}\right)+E_{11}\left(\frac{\partial^{3}w}{\partial x^{3}}-\frac{\partial^{2}\psi }{\partial x^{2}}\right)\\ =I_{0}\frac{\partial^{2}u}{\partial t^{2}}+\left(I_{3}-I_{1}\right)\frac{\partial^{3}w}{\partial x\partial t^{2}}-I_{3}\frac{\partial^{2}\psi }{\partial t^{2}}, \end{array}$$

(34) $$\begin{array}{l} \left(B_{11}-E_{11}\right)\left(\frac{\partial^{3}u}{\partial x^{3}}\right)-\left(D_{11}-F_{11}\right)\left(\frac{\partial^{4}w}{\partial x^{4}}\right)+\left(F_{11}-H_{11}\right)\left(\frac{\partial^{4}w}{\partial x^{4}}-\frac{\partial^{3}\psi }{\partial x^{3}}\right)\\ +L_{44}\left(\frac{\partial^{2}w}{\partial x^{2}}-\frac{\partial \psi }{\partial x}\right)+\left(2e_{31}V_{0}+P\right)=I_{0}\frac{\partial^{2}w}{\partial t^{2}}\\ +\left(I_{1}-I_{3}\right)\frac{\partial^{3}u}{\partial x\partial t^{2}}+\left(2I_{4}-I_{2}-I_{5}\right)\frac{\partial^{4}w}{\partial x^{2}\partial t^{2}}+\left(I_{5}-I_{4}\right)\frac{\partial^{3}\psi }{\partial x\partial t^{2}}, \end{array}$$

(35) $$\begin{array}{l} L_{44}\left(\frac{\partial w}{\partial x}-\psi \right)-E_{11}\left(\frac{\partial^{2}u}{\partial x^{2}}\right)+F_{11}\left(\frac{\partial^{3}w}{\partial x^{3}}\right)\\ -H_{11}\left(\frac{\partial^{3}w}{\partial x^{3}}-\frac{\partial^{2}\psi }{\partial x^{2}}\right)=I_{5}\frac{\partial^{2}\psi }{\partial t^{2}}-I_{3}\frac{\partial^{2}u}{\partial t^{2}}+\left(I_{4}-I_{5}\right)\frac{\partial^{3}w}{\partial x\partial t^{2}}, \end{array}$$

At the end, for investigating different cases of this beam, various boundary edges are considered which can be computed as below

▪ **Clamped–Clamped**
  (36) $$\begin{array}{lll} w=u=\psi =0, & @ & x=0\\ w=u=\psi =0. & @ & x=L \end{array}$$
▪ **Clamped–Simply**
  (37) $$\begin{array}{lll} w=u=\psi , & @ & x=0\\ w=u=M_{x}=0, & @ & x=L \end{array}$$
▪ **Simply–Simply**
  (38) $$\begin{array}{lll} w=u=M_{x}=0, & @ & x=0\\ w=u=M_{x}=0, & @ & x=L \end{array}$$

## 3. Solving Procedure

As mentioned, for solution of motion equations besides determination of DIR, DQM is employed. In the method, the various orders of the beam’s differential equations are converted to a set of algebraic equations based on weighting coefficients. In other words, with respect to this precise procedure, one derivative of a function at one contemplated separate point can be computed, utilizing the sum of the extent of function at every separate point opted out in the scope of the solution. The approximation of the derivative function can be expressed in a general form as [50,51,52]

(39) $$\frac{d^{n}f(x_{i})}{dx^{n}}=\sum_{j=1}^{N}C_{ij}^{\left(n\right)}f(x_{j})\;\;n=1,\ldots ,N-1,$$

in which f(x) refers to the function, *N* describes number of points, $x_{i}$ represents an instance point of the function scope, $f_{i}$ expresses the amount of the function at *i*th instance point and $C_{ij}$ denotes weighting coefficients. Moreover, selecting grid points, as well as weighting coefficients would be essential parameter in gaining precise consequences. Grid points can be contemplated through Chebyshev polynomials as

(40) $$x_{i}=\frac{L}{2}\left[1-\cos\left(\frac{i-1}{N_{x}-1}\right)\pi \right]\;\;i=1,\ldots ,N_{x}$$

With respect to the Chebyshev polynomials, grid points would be denser in the neighbor of boundaries. The weighting coefficients are achieved using following relation

(41) $$C_{ij}^{\left(1\right)}=\frac{L_{1}(x_{i})}{\left(x_{i}-x_{j})L_{1}(x_{j}\right)}\;\;\;\;for\;\;i\neq j,\;\;i,j=1,2,\ldots ,N$$

where

(42) $$L_{i}(x)=\prod_{j=1}^{N}\left(x_{i}-x_{j}\right).$$

With respect to DQM, motion equations in matrix are expressed form as below:

(43) $$\left(\;\left[K\right]\left\{\begin{array}{l} \left\{d_{b}\right\}\\ \left\{d_{d}\right\} \end{array}\right\}+P{\left[K\right]}_{G}\left\{\begin{array}{l} \left\{d_{b}\right\}\\ \left\{d_{d}\right\} \end{array}\right\}+\left[M\right]\left\{\begin{array}{l} \left\{{\ddot{d}}_{b}\right\}\\ \left\{{\ddot{d}}_{d}\right\} \end{array}\right\}\right)=\left\{\begin{array}{l} \left\{0\right\}\\ \left\{0\right\} \end{array}\right\},$$

where [M],[K] as well as [C] define mass matrix, stiffness matrix and damper matrix, respectively. Further, $\left\{d_{b}\right\}$ and $\left\{d_{d}\right\}$ define boundary points as well as domain points, respectively. With respect to Bolotin’s method, elements related to {Y} can be described in Fourier series having period 2T as

(44) $$\left\{Y\right\}=\sum_{k=1,3,\ldots }^{\infty }\left[{\left\{a\right\}}_{k}\sin\frac{k\omega t}{2}+{\left\{b\right\}}_{k}\cos\frac{k\omega t}{2}\right]\;,$$

In this part, by substituting Equation (44) into Equation (43) and setting the factors of cosine and sine—as well as the sum of constant terms to zero—we have

(45) $$\left|\;\left(\left[K\right]-\left(\alpha \pm \frac{\beta }{2}\right)P_{cr}{\left[K\right]}_{G}\right)-\left[M\right]\frac{\omega^{2}}{4}\right|=0\;,$$

Hence, as to gain variation of ω and DIR based on β, the mentioned relation can be solved according to eigenvalue problem.

## 4. Numerical Consequences

This part is presented to investigate and analyze the influence of diverse variables on the dynamic behavior of the beam with length of L = 2 m and thickness of h = 30 cm. As noted previously, the hypothesized beam is composed of epoxy and CFs. In addition, CNTs are considered the reinforcements and properties of these materials are assumed, according to [34]. Young’s modulus, Poisson’s ratio and density of epoxy are $E^{M}=3.51\;\mathrm{GPa}$, $\nu^{M}=0.3$ and $\rho^{M}=1200\;\mathrm{Kg}/m^{3}$, respectively. Further, material characteristics including Young’s modulus, Shear modulus, Poisson’s ratio and density related to the CFs, respectively are $E_{11}^{F}=233.05\;\mathrm{GPa}$, $E_{22}^{F}=23.1\;\mathrm{GPa}$, $G_{12}^{F}=8.96\;\mathrm{GPa}$, $\nu^{F}=0.2$ and $\rho^{F}=1750\;\mathrm{Kg}/m^{3}$. Likewise, Young’s modulus, Poisson’s ratio, density, outer diameter, thickness and length of CNT, respectively are $E^{CN}=640\;\mathrm{GPa}$, $\nu^{CN}=0.27$, $\rho^{CN}=1350\;\mathrm{Kg}/m^{3}$, $d^{CN}=1.4\;\mathrm{nm}$, $t^{CN}=0.34\;\mathrm{nm}$ and $\ell^{CN}=25\times 10^{-6}\;m$.

### 4.1. Validation

**Figure 2:**
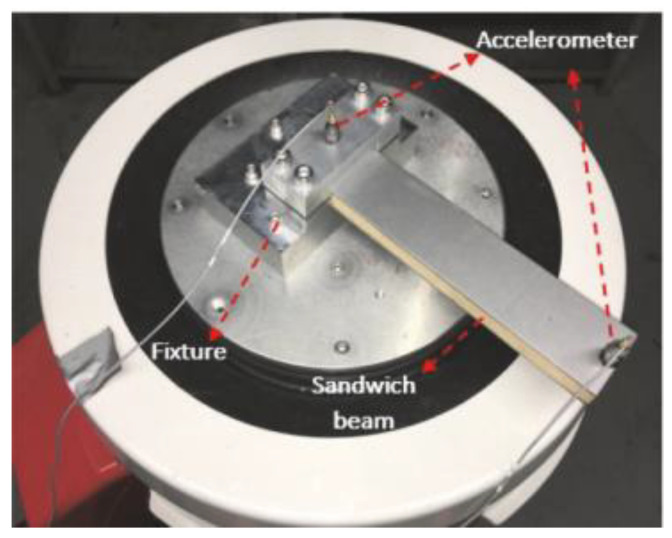
Schematic of experimental setup extracted from Joubaneh et al. [53].

To date, in spite of proliferation of researches in mechanic and material fields, there is no work analyzing dynamic behavior of hybrid nanocomposite polymer beams reinforced via CFs and CNTs using ESDBT and determining DIR. Therefore, in order to demonstrate the precision—as well as validity of the considered theories and numerical methods—some characteristics containing CNTs, CFs and ESDBT are disregarded to fit in this beam with the research published by Joubaneh et al [53]. In this research, vibration of a three-layer beam is studied based on theoretical and experimental methods. For this purpose, a beam with shear modulus of 22.1 GPa, Poisson’s ratio of 0.3, density of 60 kg/m^3^, thickness of 15 mm and length of 260 mm is assumed which is covered with two layers at the top and bottom of core with Young’s modulus of 210 GPa, Poisson’s ratio of 0.3, density of 7900 kg/m^3^ and thickness of 1.9 mm. For experimental analyses, a clamped–free boundary condition of beam with three layers is installed on a VDL shaker (B & K V830-335-SPA16K) using a head plate and a fixture, both made of aluminum, as shown in Figure 2.

The theoretical and experimental results of [53] are shown in Table 1: the outcomes are validated with our numerical method. As can be seen, the results of this study are close to the theoretical and experimental results [53,54].

In another validation, the buckling of nanocomposite beam is studied. A Poly methyl methacrylate (PMMA) with Young’s modulus, Poisson’s ratio and density of epoxy are $E^{M}=2.5\;\mathrm{GPa}$, $\nu^{M}=0.3$ and $\rho^{M}=1190\;\mathrm{Kg}/m^{3}$, respectively is assumed which is reinforced with 0.12 carbon nanotube with Young’s modulus, Poisson’s ratio and the density of epoxy are $E_{11}^{CN}=600\;\mathrm{GPa},\;E_{22}^{CN}=10\;\mathrm{GPa}$, $\nu^{CN}=0.19$ and $\rho^{CN}=1400\;\mathrm{Kg}/m^{3}$, respectively. The non-dimensional buckling load $\bar{P}=P/hE^{M}$ for different boundary condition is presented in Table 2. It is shown that the result of this study are in good agreement with Asadi and Wang [55] and Yas and Samadi [56].

Table 3 presents the dimensionless frequency of the beam reinforced by CNTs, considering the material properties the same as those mentioned in before validation. The results are reported for different theories of first-order shear deformation theory (FSDT), third order shear deformation theory (TSDT), exponential order shear deformation theory (ESDT), higher order shear deformation theory (HSDT) and trigonometric shear deformation theory (TrSDT) which are presented by Wattanasakulpong and Ungbhakorn [57]. It is seen that the accuracy of the obtained results is good.

### 4.2. Parametric Study

In this section, the dynamic stability of the structure is studied for different parameters. All of figures show the excitation frequency against dynamic load factor (i.e., β) which represent the DIR of structure. In these figures, the regions inside and outside the boundary curves correspond to unstable (parametric resonance) and stable regions, respectively.

**Figure 3:**
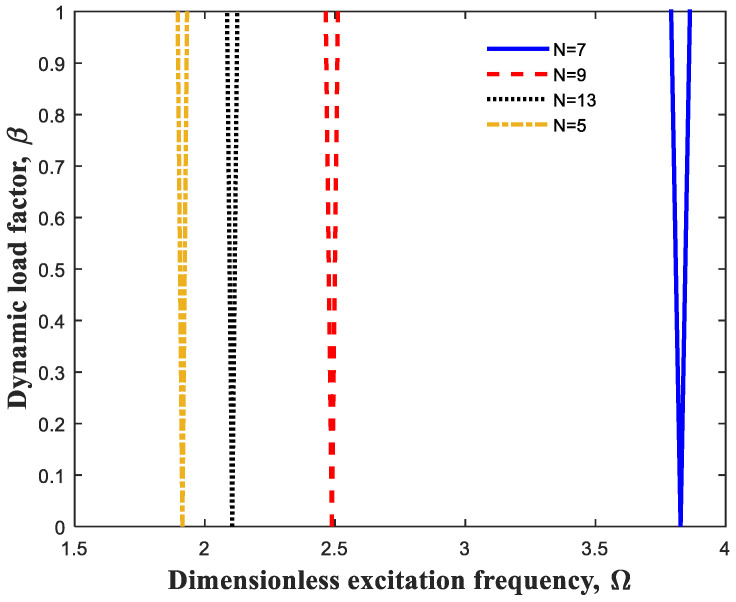
Precision and convergence of differential quadrature method (DQM).

At first, so as to assess the precision as well as convergence of applied numerical method, Figure 3 is depicted. As observed, excitation frequencies were obtained for various grid points. It is clear that fifteen grid points can be factored in the precise outcomes in this numerical method.

**Figure 4:**
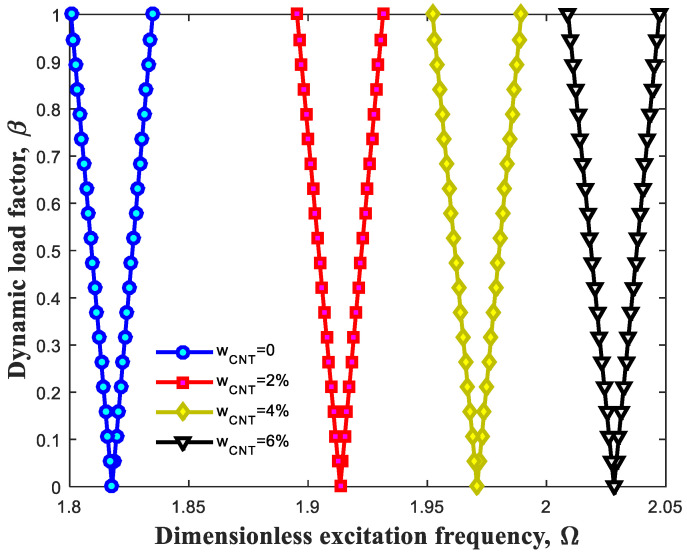
Effect of CNT weight percentage on dynamic instability region (DIR).

Figure 4 presents the influence of CNT weight-percentage on DIR. Likewise, it is worth noting that the inside sections of triangular shapes represent unstable areas and consequently, the outside sections indicate stable areas of this beam. It is obvious that rise of CNT weight percentage triggers increase in excitation frequency of this structure. To put it differently, the DIR takes place at higher extents of excitation frequencies while increasing weight percentage of CNTs. It is justified with this fact that increase in CNT weight percentage leads to enhancement of stiffness as well as bending rigidity related to this structure.

**Figure 5:**
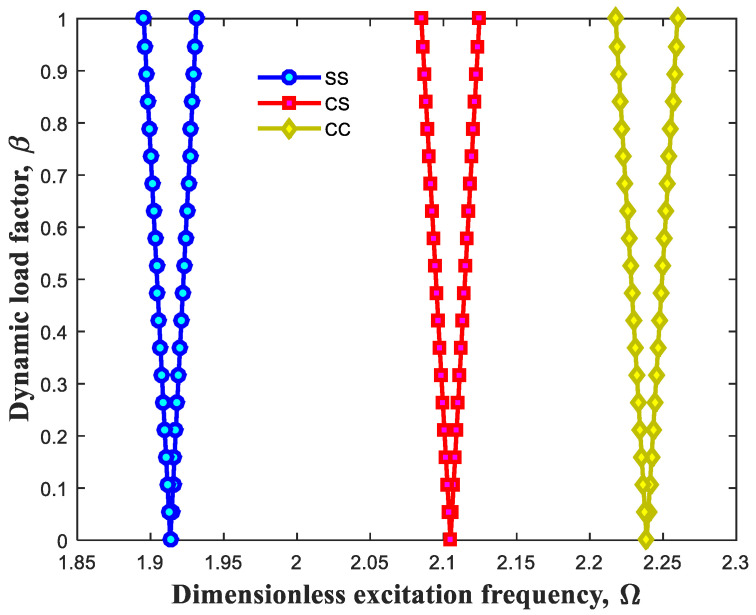
Effect of distinct boundary edges on DIR.

In this work, various boundary edges were factored in to extend this research for different cases. Therefore, Figure 5 shows the influence of different boundary edges containing clamped–clamped (CC), clamped–simply (CS) and simply–simply (SS) boundary edges on the dynamic behaviors of this system. As observed, boundary edges have an undisputed effect on the DIR of the beam, and it is vivid that this structure with CC boundary edges contains higher DIR compared to CS as well as SS having lower quantities. In fact, it raises bending rigidity.

**Figure 6:**
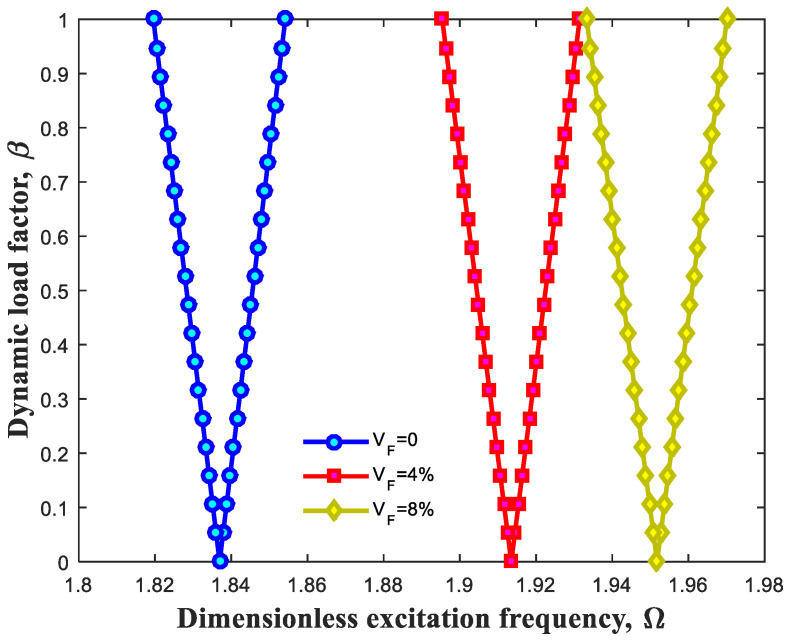
Effect of volume percent of CFs on DIR.

Figure 6 illustrates to indicate the significance related to presence of CFs and its volume percent and consequently its influence on the dynamic behaviors of the system. As expected, increase in volume percent of CFs results in shift of DIR to higher extents of excitation frequencies owing to rise of system’s stiffness.

**Figure 7:**
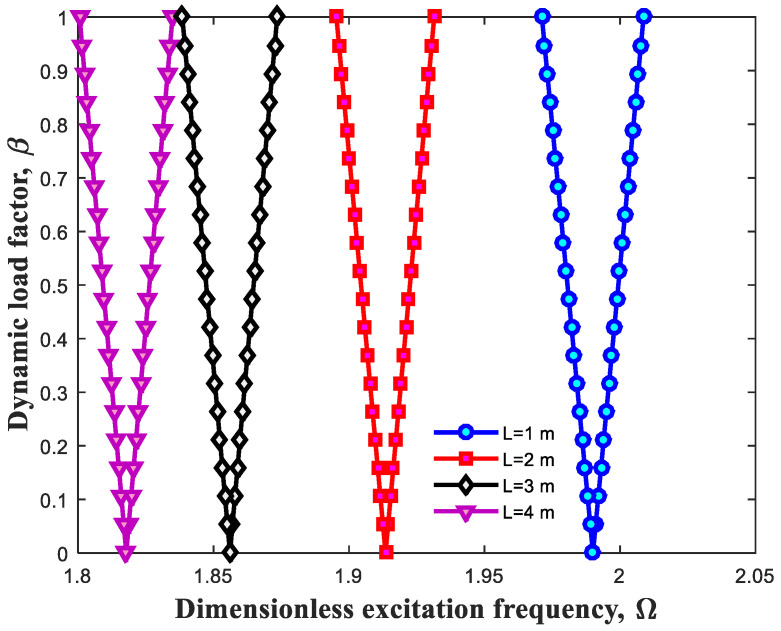
Effect of beam length on DIR.

**Figure 8:**
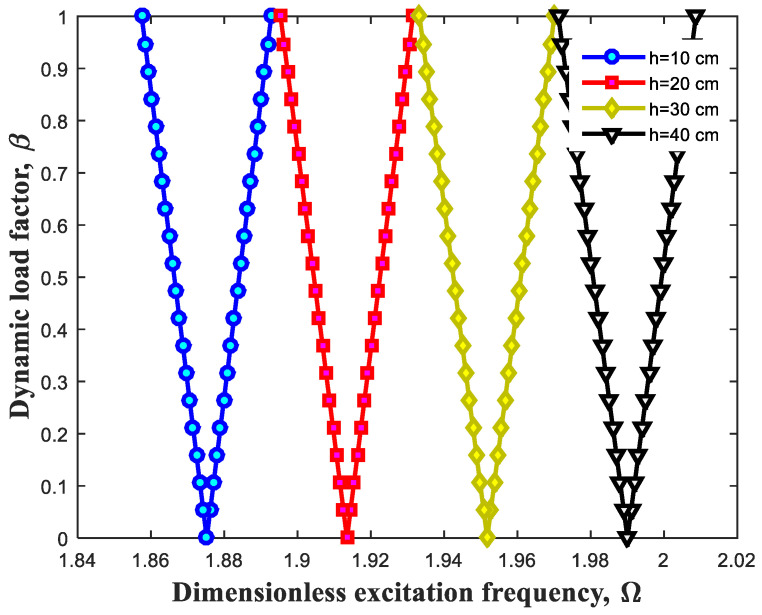
Effect of beam thickness on DIR.

Having been plotted below, Figure 7 and Figure 8 are the exemplary states to ascertain the influence of geometric variables on the dynamic behavior of the beam. Figure 7 and Figure 8, respectively evaluate the changes of length and thickness of structure. It is construed that rise of length parameter of polymeric beam results in alteration of DIR into the lower extents. In fact, this change can affect characteristics related to the structure and cause softness of whole structure. Inversely, increment of the thickness leads to improvement in the stiffness of system and move in DIR to the higher extents.

## 5. Conclusions

This concerned the evaluation of dynamic instability related to hybrid nanocomposite polymer beam. The considered beam composed by multiphase nanocomposite, include polymer-CNTs- CFs. Halpin–Tsai as well as micromechanics equations were applied to compute effective material characteristics correlative to multiphase nanocomposite layers. Employing ESDBT besides Hamilton’s principle, governing equations were obtained. Further, the DQM and Bolotin procedures were utilized to solve the governing equations and achieve dynamic behavior of polymer beam. Different parameters such as various boundary edges, geometric parameters, CNTs and CFs volume fractions were brought up and their influences were illustrated on DIR. The prominent outcomes are presented as following

- Increments of weight-percentage of CNTs can lead to shift of DIR to the right;
- Presence of CFs as well as CNTs play paramount role in dynamic behavior of hybrid polymer structure and raise the excitation frequency;
- Considering CC boundary edge causes increase in excitation frequency of structure compared to CS and SS boundary edges;
- The obtained results accentuate the geometric parameters and their influence on the dynamic behavior of structures.

## Figures and Tables

**Table 1:** Comparison of the obtained natural frequencies.

Mode	DQ Method [54]	Experiment [53]	Present Work
1	118.00	111.19	112.12
2	381.00	364.60	366.23
3	719.84	680.80	687.33
4	1141.94	1063.50	1074.98
5	1676.51	1652.07	1666.44

**Table 2:** Comparison of the obtained critical buckling load.

BC	Asadi and Wang [55]	Yas and Samadi [56]	Present Work
SS	0.09831	0.09859	0.09844
CS	0.14878	0.14948	0.14852
CC	0.21264	0.21395	0.21272

**Table 3:** Comparison of the obtained dimensionless frequency.

Theory	Wattanasakulpong and Ungbhakorn [57]	Present Work
FSDT	0.9976	0.9974
TSDT	0.9745	0.9749
ESDT	0.9756	0.9759
HSDT	0.9745	0.9744
TrSDT	0.9749	0.9741

## Data Availability

The data presented in this study are available on request from the corresponding author.

## Appendix A

(A1) $$N_{x}=A_{11}\left(\frac{\partial u}{\partial x}+\frac{1}{2}{\left(\frac{\partial w}{\partial x}\right)}^{2}\right)-B_{11}\left(\frac{\partial^{2}w}{\partial x^{2}}\right)+E_{11}\left(\frac{\partial^{2}w}{\partial x^{2}}-\frac{\partial \psi }{\partial x}\right),$$

(A2) $$M_{x}=B_{11}\left(\frac{\partial u}{\partial x}+\frac{1}{2}{\left(\frac{\partial w}{\partial x}\right)}^{2}\right)-D_{11}\left(\frac{\partial^{2}w}{\partial x^{2}}\right)+F_{11}\left(\frac{\partial^{2}w}{\partial x^{2}}-\frac{\partial \psi }{\partial x}\right),$$

(A3) $$F_{x}=E_{11}\left(\frac{\partial u}{\partial x}+\frac{1}{2}{\left(\frac{\partial w}{\partial x}\right)}^{2}\right)-F_{11}\left(\frac{\partial^{2}w}{\partial x^{2}}\right)+H_{11}\left(\frac{\partial^{2}w}{\partial x^{2}}-\frac{\partial \psi }{\partial x}\right),$$

(A4) $$Q_{x}=L_{44}\left(\frac{\partial w}{\partial x}-\psi \right),$$

in which

(A5) $$A_{11}=\int_{}C_{11}\;dA,$$

(A6) $$B_{11}=\int_{}C_{11}\;zdA,$$

(A7) $$E_{11}=\int_{}C_{11}\;\Phi (z)dA,$$

(A8) $$F_{11}=\int_{}C_{11}\;z\Phi (z)dA,$$

(A9) $$H_{11}=\int_{}C_{11}\;\Phi {\left(z\right)}^{2}dA,$$

(A10) $$L_{44}=\int_{}C_{44}\;\frac{\partial \Phi (z)}{\partial z}dA,$$
